# Editorial: Year in review: discussions in genetics of aging

**DOI:** 10.3389/fgene.2024.1470451

**Published:** 2024-09-17

**Authors:** Blanka Rogina, Claudio Franceschi, Heidi A. Tissenbaum

**Affiliations:** ^1^ Department of Genetics and Genome Sciences, School of Medicine, University of Connecticut Health Center, Farmington, CT, United States; ^2^ Institute for Systems Genomics, Farmington, CT, United States; ^3^ Laboratory of Systems Medicine of Healthy Aging, Institute of Biogerontology, Lobachevsky University, Nizhny Novgorod, Russia; ^4^ Department of Molecular, Cell, and Cancer Biology, UMass Chan Medical School, Worcester, MA, United States

**Keywords:** genetics of aging, epigenetic clocks, biomarkers, accelerated biological aging, multi-omics, single-cell RNA, COVID-19

This Editorial is for the Research Topic with the title “*Year in review: discussions in genetics of aging*”. This Research Topic is a compilation of original articles, opinion articles and a mini-review inspired by the most viewed articles published in 2022 (Mao et al., 2022; Ahlers et al., 2022; Pang et al., 2022; Kuzub et al., 2022; Chen et al., 2022). The focuses of these 2022’s manuscripts were cutting edge genomic and genetics studies describing timely topics such as senescence, COVID-19, and aging clocks. Briefly, Mao et al. (2022) used integrative analysis of transcriptome, and proteome of young and old senescence fibroblasts and identified four senescence related molecules (SRMs) (Mao et al., 2022). The four SRMs that could potentially be used as biomarkers of human skin aging and include: ETF1, which may be affecting inflammatory factors, NGKB1, which regulates immune response, MOXD1, which affects the WNT pathway, and ASAH1, which may affect aging via its effects on cell cycle-related genes (Mao et al., 2022). In the second manuscript, Chen et al. (2022) reviewed the preclinical and clinical efficacies of dietary supplements and natural products and how dietary supplements and natural products may promote healthspan. The manuscript by Chen et al. (2022) also discussed current molecular mechanisms underlying the aging processes related to dietary supplements and natural products dietary supplements (Chen et al., 2022). The third manuscript by Pang et al. (2022) examined a longitudinal study of DNA methylation and epi-genetics, and their association with SARS-CoV-2 infection and mRNA after COVID-19 vaccination (Pang et al., 2022). The authors reported age-associated effects on clocks prior and after confirmation of COVID-19 infection, with epigenetic clock estimates of PhenoAge and GrimAge being significantly increased in individuals over 50 years of age, and decreased in individuals younger than 50, following infection. The longitudinal study also revealed benefits of mRNA based COVID-19 vaccination, illustrated by reduced epigenetic clocks following vaccination in individuals over 50. Epigenetic age was also studied in the fourth manuscript by Kuzub et al. (2022), where the authors evaluated the Ukrainian population based on CpG methylation levels and two different epigenetic clocks based on the 4 CpG and 2 CpG sites in the human genome (Kuzub et al., 2022). Both models examined in the study show good age predictive accuracy. In the fifth manuscript, Ahlers et al. (2022) used single-cell RNA profiling of human skin and provided evidences that age-associated loss of the dermal sheath stem cells contributed to the aging skin phenotype (Ahlers et al., 2022). These manuscripts attracted the most attention throughout the year and have been already referenced in a number of published manuscripts.

Aging is characterized by a progressive decline in physiological function, and impaired repair leading to a decrease in homeostasis, increased risk of diseases, and ultimately death (Helfand and Rogina, 2003). There has been an ongoing search for aging biomarkers that would define physiological function and health of an organism. Such biomarkers could then be used for monitoring progress of aging-related disorders, success of different disease treatments and/or treatments that are designed to slow down age-related decline.

There are many physiological signs of the aging process including increased physical weakness and increased risk of cardiovascular disease. To gain mechanistic insight into these biological processes, Qian et al., 2022 used a genome-wide association study (GWAS) to determine a link between 486 metabolites and frailty, sarcopenia, arterial stiffness, atherosclerosis, aortic aneurysm and peripheral vascular disease (PAD). The authors identified causative association between six metabolites and sarcopenia, PAD and atherosclerosis. In addition, they also identified 13 metabolic pathways significantly related in six age-related diseases. These six metabolites have potential to be used as biomarkers to help with identification and monitoring the treatment of age-associated increases in frailty, sarcopenia and vascular diseases.

Esterly and Zapata wrote an opinion article in which they discussed current challenges to define senescence. The authors reviewed work by Mao et al., who combined transcriptomic and proteomic approaches and identified four biomarkers of senescence in skin fibroblasts (Mao et al., 2022). They also dicussed an effort by the Cellular Senescence Network (SenNet) created in 2021 (SenNet Consortium et al., 2022), to standardize senescent cell identification (Saul et al., 2022; SenNet Consortium et al., 2022). The major goal of SenNet is to characterise senescence cells across the human lifespan using different tissues types. The group also reported identification of a gene set, named SenMayo, which consist of 125 genes enriched in senescent and SAPS-secreting human and murine cells, which resulted from 15 previously existing datasets.

Effects of COVID-19 on epigenetics clocks described by (Pang et al., 2022) inspired an original manuscript by Wang et al. The authors discussed the role of cellular senescence in COVID-19 inhibition of spermatogenesis by comparing testicular bulk-RNA-Seq data from COVID-19 patients and healthy controls. One of the top 10 KEGG enrichment pathways within the upregulated genes in COVID-19 infected patients was the senescence pathway. The authors also identified the MAPK signaling pathway as a potential mediator of COVID-19 induced testicular senescence, via its negative effects on metabolic pathways required for normal spermatogenesis. They suggest drugs targeting the MAPK pathway could block the damage to spermatogenesis by COVID-19.

Rogina and Tissenbaum provided a review on the current knowledge of the role of SIRT1 and resveratrol in diseases and aging. While this review was inspired by an inclusive review by Chen et al. review, Rogina and Tissenbaum provided a more detailed review of the original studies to identify SIRT1 as well as the trials using SIRT1 activators and inhibitors (Chen et al., 2022). SIRT1 is a member of a highly conserved family of histone deacetylases. SIRT1, as an epigenetic regulator, has effects on a variety of processes including metabolism, stress response, protein synthesis, circadian rhythm, genomic stability, replicative senescence, among others. Disregulation of SIRT1 has been associated with some diseases. As a result, novel SIRT1 activators and inhibitors have been used in a number of clinical studies for treatment of metabolic diseases, coronary heart disease, cancer, and neurodegenerative diseases.

Karagiannis et al., wrote an opinion article inspired by Ahlers et al., 2022; (Ahlers et al., 2022). Karagiannis et al., provided a new perspective on the Bayesian differential analysis of cell type proportions. They analyzed single cell distribution data by using a Bayesian multinomial regression model, which takes into account cell type proportional compositional constrains and does not require a reference cell type. In addition, the new approach estimates the absolute proportions of cell type per sample, which are easier to interpret compared to odds ratios or other metrics.

The studies included in this Research Topic, provide a framework for future studies that can contribute to our knowledge on identification, monitoring and treatment of age-related disease.
